# Mobile Efficient Diagnostics of Infectious Diseases via On‐Chip RT‐qPCR: MEDIC‐PCR

**DOI:** 10.1002/advs.202302072

**Published:** 2023-08-16

**Authors:** Kiran Shrestha, Seongryeong Kim, Jiyeon Han, Gabriela Morales Florez, Han Truong, Trung Hoang, Sajjan Parajuli, Tiara AM, Beomsoo Kim, Younsu Jung, Abdurhaman Teyib Abafogi, Yugyeong Lee, Seung Hyun Song, Jinkee Lee, Sungsu Park, Minhee Kang, Hee Jae Huh, Gyoujin Cho, Luke P. Lee

**Affiliations:** ^1^ Department of Biophysics Institute of Quantum Biology Sungkyunkwan University Suwon 16419 South Korea; ^2^ Department of Intelligent Precision Healthcare Convergence Sungkyunkwan University Suwon 16419 South Korea; ^3^ Department of Biological Science College of Science Sungkyunkwan University Suwon 16419 South Korea; ^4^ Research Engineering Center for R2R Printed Flexible Computer Sungkyunkwan University Suwon 16419 South Korea; ^5^ School of Electronic and Electrical Engineering Sungkyunkwan University Suwon 16419 South Korea; ^6^ School of Mechanical Engineering Sungkyunkwan University Suwon 16419 South Korea; ^7^ Department of Biomedical Engineering Sungkyunkwan University Suwon 16419 South Korea; ^8^ Department of Electronics Engineering Sookmyung Women's University Seoul 04310 South Korea; ^9^ Biomedical Engineering Research Center Smart Healthcare Research Institute Samsung Medical Center Seoul 06352 South Korea; ^10^ Department of Medical Device Management and Research SAIHST (Samsung Advanced Institute for Health Sciences & Technology) Sungkyunkwan University Seoul 06355 South Korea; ^11^ School of Medicine Department of Laboratory Medicine and Genetics Samsung Medical Center Sungkyunkwan University Seoul 06351 South Korea; ^12^ Harvard Medical School Department of Medicine Brigham Women's Hospital Boston MA 02115 USA; ^13^ Department of Bioengineering University of California at Berkeley Berkeley CA 94720 USA; ^14^ Department of Electrical Engineering and Computer Science University of California at Berkeley Berkeley CA 94720 USA

**Keywords:** covid‐19, diagnostics, low‐cost, rapid, rt‐qpcr

## Abstract

The COVID‐19 outbreak has caused public and global health crises. However, the lack of on‐site fast, reliable, sensitive, and low‐cost reverse transcription polymerase chain reaction (RT‐PCR) testing limits early detection, timely isolation, and epidemic prevention and control. Here, the authors report a rapid **m**obile **e**fficient **d**iagnostics of **i**nfectious diseases via on‐**c**hip ‐RT‐quantitative PCR (RT‐qPCR): MEDIC‐PCR. First, the authors use a roll‐to‐roll printing process to accomplish low‐cost carbon‐black‐based disposable PCR chips that enable rapid LED‐induced photothermal PCR cycles. The MEDIC‐PCR can perform RT (3 min), and PCR (9 min) steps. Further, the cohort of 89 COVID‐19 and 103 non‐COVID‐19 patients testing is completed by the MEDIC‐PCR to show excellent diagnostic accuracy of 97%, sensitivity of 94%, and specificity of 98%. This MEDIC‐PCR can contribute to the preventive global health in the face of a future pandemic.

## Graphical abstract

The highly efficient light‐to‐heat conversion is mediated by multi‐layered graphene and amorphous carbon heterostructure thin film layer via induced electron‐phonon and phonon‐phonon couplings upon irradiation of a NIR light. MEDIC‐PCR achieved ultra‐fast (<15 min) RT‐qPCR with excellent diagnostic accuracy of 97%, sensitivity of 94%, and specificity of 98%.

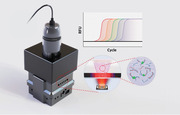

## 1 Introduction

Since the emergence of the coronavirus disease 2019 (COVID‐19) pandemic, there has been an acute need felt for reliable and accurate nucleic acid‐based molecular diagnostic devices^[^ ^1^, ^2^ ^]^ for the timely assessment of treatment and isolation of early‐stage,^[^ ^3^ ^]^ asymptomatic patients.^[^ ^4^, ^5^ ^]^ For these devices to be effective, they must be mobile, inexpensive, rapid, and simple to operate,^[^ ^6^, ^7^ ^]^ all the while maintaining the accuracy and sensitivity that conventional polymerase chain reaction (PCR)^[^ ^8^ ^]^ tests provide. Infectious diseases can be diagnosed in various ways other than PCR, such as loop‐mediated isothermal amplification (LAMP),^[^ ^9^, ^10^ ^]^ recombinase polymerase amplification (RPA),^[^ ^11^ ^]^ and rolling circle amplification (RCA).^[^ ^12^, ^13^ ^]^ LAMP has high sensitivity, facilitates cheaper, and faster testing,^[^ ^14^, ^15^, ^16^ ^]^ but with a significant limitation: its high false‐positive rate due to the high non‐specific amplification.^[^ ^17^ ^]^ Similarly, RPA method is advantageous mainly because of its speed, simplicity, and selectivity,^[^ ^18^ ^]^ but it mandates purification and protein design steps afterward.^[^ ^17^ ^]^ Also, this method poses high chances of non‐specific amplification. RCA has broad applications in biology, science, and medicine due to its simplicity but challenges in sample purification, reaction time, mass production, and shelf storage time.^[^ ^12^, ^19^ ^]^ Next, the lateral flow assay (LFA) has high speed, specificity, and simplicity.^[^ ^20^, ^21^ ^]^ Still, it has extremely poor sensitivity as the test is based on detecting the antibody response to an infection and viral antigens.^[^ ^22^ ^]^ Although these nucleic acid amplification tests (NAAT) and LFAs are simpler and speedier, the gold standard PCR technique provides better accuracy, sensitivity, and specificity. Thus, worldwide efforts have been poured into developing such portable and fast quantitative reverse transcriptase PCR (RT‐qPCR) as a point‐of‐care (POC) device.^[^ ^23^, ^24^, ^25^, ^26^ ^]^ To render RT‐qPCR as the POC device, the main challenge remains to condense the bulky instrument into a hand‐held, palm‐sized device that the general population can use in a simple, inexpensive, and rapid manner while the accuracy and sensitivity remain uncompromised.

To fulfill RT‐qPCR in the POC device for diagnosing COVID‐19, a light‐induced photothermal cycler^[^ ^27^, ^28^, ^29^ ^]^ in which the light‐to‐heat conversion can be carried out via plasmonic resonance.^[^ ^30^, ^31^ ^]^ In addition, the long workflow time^[^ ^32^, ^33^ ^]^ and the requirement of the medical professionals for the sample preparation process^[^ ^34^ ^]^ should be eliminated so that the RNA extraction‐free lysate can be directly processed in the RT‐qPCR, called a direct RT‐qPCR.^[^ ^35^ ^]^ In the direct RT‐qPCR, utilization of organic molecules with an amine group or an ionic surfactant can lyse the severe acute respiratory syndrome coronavirus 2 (SARS‐CoV‐2)^[^ ^36^ ^]^ and flu viruses^[^ ^37^ ^]^ without inhibition in the reverse transcription and the DNA amplification (PCR), all the while maintaining good accuracy, sensitivity, and specificity for the target virus. Although the time required for the thermal cycles and the sample preparation was dramatically reduced by implementing both the plasmonic photothermal cycler^[^ ^28^, ^38^ ^]^ and the direct PCR in the RT‐qPCR, the cost per PCR test can still be reduced by innovating the materials and manufacturing techniques. To reduce the cost per PCR close to LFAs, a film of low‐cost photothermal material like carbon^[^ ^39^, ^40^ ^]^ can be manufactured with high throughput manufacturing techniques such as the roll‐to‐roll (R2R) printing method.^[^ ^41^, ^42^ ^]^ Therefore, to perform RT‐qPCR in the POC device, the assay would be beneficial if it is achieved in a single device within tens of minutes by utilizing lysate without the RNA purification and the photothermal cycler with the inexpensive photothermal heating and cooling method. Thus, the photothermal cycler with the affordable heating and cooling process and the direct RT‐qPCR without the RNA purification should be combined into a single PCR device for the real‐time RT‐qPCR in the POC device. Having reagents cost included to lower the cost of the PCR test and to deliver during the high demands, the production of PCR chip should be easily scalable, and it should utilize abundant material and a high‐throughput manufacturing method. As for being POC, the device should be assembled with the minimum hardware to fit in a palm including a camera system to monitor the fluorescence intensity of the PCR reagent to determine the amplification curve and cycle threshold (*C_t_*) value^[^ ^43^ ^]^ in real‐time. Therefore, the cost and time per test can be dramatically reduced by employing the high‐throughput manufacturing method^[^ ^44^ ^]^ and inexpensive materials^[^ ^45^ ^]^ to fabricate the disposable PCR chip.^[^ ^46^ ^]^

**Figure 1:**
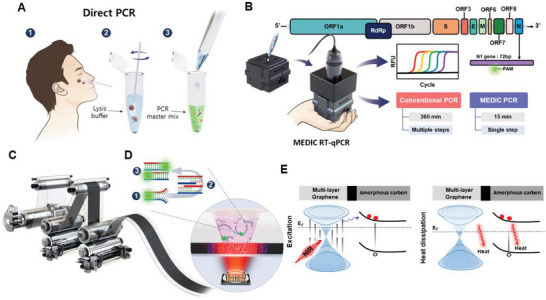
MEDIC‐PCR. A) Simplified sample preparation steps: The nasopharyngeal swab was transferred to the PCR tube containing lysis buffer, and then, after stirring gently, PCR reagent was added to prepare the final solution for the RT‐qPCR. B) Sample was loaded to the MEDIC‐PCR using a pipette. The MEDIC‐PCR was optimized to amplify 72 bases paired N1 gene of SARS‐CoV‐2 recommended by the CDC using a real‐time RT‐qPCR. C) Roll‐to‐roll gravure and imprinter were used to print carbon‐black thin film and consequently in‐line imprinted PDMS mini‐well as a disposable PCR chip with high‐throughput and low production cost. D) Cross sectional illustration of the PCR chip during the PCR thermal cycle. The near‐IR light from LED was converted to thermal energy by the printed carbon‐black film used for photothermal cycling. E)The thermal energy required for RT‐qPCR was harnessed by utilizing printed carbon‐black (multi‐layered graphene and amorphous carbon heterostructure) thin film as a photothermal converter, and near‐IR (940 nm) LED was used as the source of the light for generating electron‐phonon and phonon‐phonon couplings which led to a thermal conversion efficiency above 100%.

In this study, we report the development of a portable palm‐sized POC device, which eliminated a conventional sample preparation method and used a simplified sample preparation method based on direct PCR (**Figure** **1**a). The sample swab is placed inside the tube, containing a preloaded lysis buffer. And then, the lysed solution is loaded in a tube with a “direct PCR master mix”. And we used calibrated pipettes to dispense 1 µl from the prepared PCR solution to the mini‐well. The disposable PCR chip for Mobile Efficient Diagnostics of Infectious diseases via on‐Chip RT‐qPCR (MEDIC‐PCR) using photothermal cycler to amplify the COVID‐19 N1 gene (Figure 1b) by the general population at home. Our MEDIC‐PCR can carry out real‐time RT‐qPCR via the disposable PCR chip. The total turnaround time for the PCR diagnosis with the MEDIC‐PCR was less than 15 min, and sample‐to‐answer was achieved with single‐step simplified sample preparation for direct PCR. As the typical high‐throughput manufacturing method, the carbon‐black thin film was printed on the polyethylene terephthalate (PET) substrate due to its low autofluorescence^[^ ^47^ ^]^ by using a R2R gravure technique. Then polydimethylsiloxane (PDMS)‐based millimeter‐sized wells called, mini‐wells were in‐line imprinted over the printed carbon‐black layer, collectively called as the PCR chip (Figure 1c). Using the MEDIC‐PCR, we amplified and detected the SARS‐CoV‐2 reference RNA within 15 min (Figure 1d). In the MEDIC‐PCR, the light‐to‐heat conversion was mediated by carbon‐black (multi‐layered graphene and amorphous carbon hybrid) thin film layer via induced electron‐phonon and phonon‐phonon couplings^[^ ^48^, ^49^, ^50^, ^51^ ^]^ upon irradiation of a near‐infrared (NIR) light (Figure 1e). We further validated the MEDIC‐PCR by testing nasopharyngeal samples from the cohort of 89 COVID‐19 and 103 non‐COVID‐19 patients with a sensitivity of 94% and specificity of 98% for the N1 gene of SARS‐CoV‐2. Remarkably, MEDIC‐PCR achieved an excellent diagnostic accuracy of 97%, proving the device's immediate efficacy in practical deployment.

## 2 Results

### 2.1 R2R Gravure and Imprinter for the Mass Production of PCR Chips

**Figure 2:**
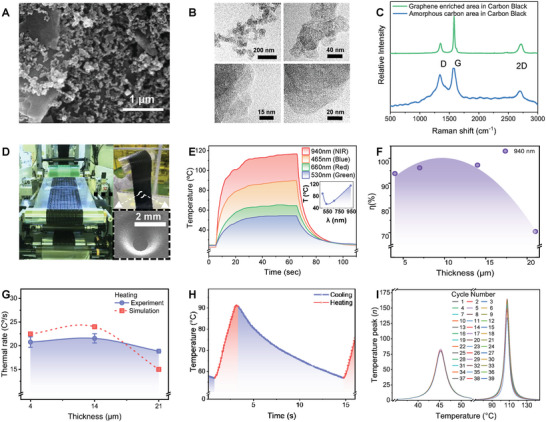
Characterization of R2R gravure printed carbon‐black film and imprinted PDMS wells for photonic PCR chip. A) SEM image of R2R gravure printed carbon‐black thin film. The amorphous/unstructured carbon was agglomerated spherical particles with graphite flakes. B) TEM images of a detailed structure of agglomerated carbon‐black particles showing multi‐layered graphene and amorphous carbon heterostructure. C) Raman spectra of multi‐layered graphene and amorphous carbon heterostructure of the printed carbon‐black thin‐film. The position of the G (1600 cm^−1^) peak and the spectral features of the 2D band at 2700 cm^−1^ confirm that carbon‐black has multi‐layered graphene and amorphous carbon. D) R2R gravure printed and imprinted PCR chip array using room temperature curable PDMS (left image). The resulting disposable PCR chips with the SEM image of an individual PCR chip (right bottom image). E) Thermal performance of the printed carbon‐black thin film with the thickness of 14 µm excited with a constant power of 465, 530, 660, and 940 nm of the LED light. The arbitrary plot of the area under the curve (inset) assisted in visualizing the heating rate during 60 s of LED light irradiation. The test was performed using 5 µL of a solution in the PCR chip. F) Photothermal efficiency of printed carbon‐black thin films with thicknesses of 4, 7, 14, and 21 µm, respectively, by irradiating 940 nm wavelength of the LED light. The highest efficiency was 97.3% at 14 µm thickness, selected for further characterization and application. G) Thermal rates (heating) attained by simulation using ANSYS FLUENT (Version‐2019 R2, ANSYS, Inc.) and experiment using the MEDIC‐PCR thermal cycler with different thicknesses of the printed carbon‐black thin films. H) A typical single heating and cooling step among 40 thermal cycles using a PCR chip in the MEDIC‐PCR. I) Photothermal conversion stability of the R2R manufactured PCR chips. The distribution graph showed the calibration of annealing and denaturation temperatures of 40 thermal cycles from 355 individual PCR chips.

To accomplish photothermal cyclers through inexpensive and scalable materials, electron‐rich, chemically, and physically stable carbon‐black^[^ ^52^ ^]^ is the right candidate to replace novel metals since the interaction of the incident photons with a high number of *π* electrons in the carbon‐black^[^ ^53^ ^]^ dissipates a considerable magnitude of thermal energy.^[^ ^54^, ^55^ ^]^ The electronic transitions and resonance in the oscillation of *π* electron clouds by the irradiating light (incident photons)^[^ ^56^ ^]^ result in the vibrational transitions^[^ ^57^ ^]^ allowing electron‐phonon and phonon‐phonon coupling in the carbon‐black.^[^ ^49^, ^50^, ^51^ ^]^ Since the carbon‐black has a hetero‐structure of multi‐layered graphene and amorphous carbon (**Figure** **2**a,b), the amorphous carbon allows breathing oscillations^[^ ^58^, ^59^ ^]^ and it increases electron‐phonon generation raising the efficiency of the photothermal conversion.^[^ ^60^, ^61^ ^]^ We observed an interesting phenomenon while Raman spectroscopy of the printed carbon‐black film was measured (Figure 2c). We observed shiny and non‐shiny spots within the same sample, and a smaller Raman peak was observed in the 2D band (2700 cm^−1^) than in the G band (1600 cm^−1^), which proves the multilayer graphene^[^ ^62^, ^63^ ^]^ enriched areas in the carbon black. Similarly, the Raman peak observed at the non‐shiny site shows nearly equal G (1300 cm^−1^) and D band,^[^ ^64^ ^]^ which indicates the existence of amorphous carbon in the printed film proving carbon‐black to be a hybrid material of multi‐layer of graphene and amorphous carbon (Figure S1, Supporting Information). The high photothermal conversion efficiency,^[^ ^60^ ^]^ easy ink formulatibility ,^[^ ^52^, ^65^ ^]^ and low carbon‐black cost are beneficial to fabricating efficient photothermal devices by using the high‐throughput manufacturing method to innovate a thermal cycler in the MEDIC‐PCR.

As a high‐throughput manufacturing method, R2R gravure and imprinter were integrated for a continuous in‐line additive manufacturing system (Figure S2a, Supporting Information) to print carbon‐black on the PET substrate (Figure S2b, Supporting Information) and consequently imprinted polymer‐based mini‐wells by flexographic plate as an imprinting mold (Figure S2c, Supporting Information) to completely fabricate disposable PCR chips (Figure 2d; Figure S2d,e, Supporting Information). We first optimized the thickness of the printed carbon‐black to obtain the highest photothermal conversion efficiency. The carbon‐black thin films with the thickness of 4, 7, 14, and 21 µm were respectively printed by the R2R gravure on the PET film with a thickness of 100 µm at a printing speed of 5.4 m min^−1^, printed by the R2R gravure on the PET film with a 100 µm at a printing speed of 5.4 m min^−1^, yielding a 6480 cm^2^ min^−1^ photothermal area. Since the carbon‐black has a unique property as a strong absorber of light over a broad range of wavelengths beyond the visible range,^[^ ^54^ ^]^ we examined to find out the best resonating wavelength of a LED light for the maximum efficiency in the photothermal conversion (wavelengths ranging from 465 to 940 nm) with fixed optical power (170 mW) (Table S1, Supporting Information). This work selected a wavelength of 940 nm as the efficient light‐heat conversion since the power transfer reached the maximum efficiency with the printed carbon‐black thin film of a thickness of 14 µm (Figure 2e), and no interference by fluorescence reporters was observed.^[^ ^66^ ^]^ The inset plot in Figure 2e showed that the highest temperature was reached at 940 nm wavelength by exposing the LED light for 60 s with the excitation sources of the same power (170 mW). However, carbon‐black ink showed no particularly strong resonance peak, but a flat response along the range of near‐IR wavelength (Figure S3, Supporting Information). Based on the selected wavelength of 940 nm, the photothermal conversion efficiency of the printed carbon‐black film was calculated using the equation of the photothermal conversion efficiency,^[^ ^67^ ^]^ $\eta \;=\frac{Q}{Q_{total}}\;=\frac{C_{p}*m*▵T}{I*S*t}$, where *C*_p_ is the specific heat capacity, *m* is the mass, *ΔT* is the temperature change, *I* is the intensity of the incident light, *S* is the effective area, and *t* is the response time. The photothermal conversion efficiencies were calculated respectively for 4, 7, 14, and 21 µm thicknesses of printed carbon‐black thin films. Utilizing the experimental parameters of the printed carbon‐black thin films (Figure S4 and Table S2 Supporting Information) and the equation of the photothermal conversion efficiency, the highest conversion efficiency was calculated for the film with a thickness of 14 µm when illuminated by a 940 nm (3.75 W) wavelength. The photothermal efficiency decreases as thickness increases due to higher thermal mass and generation of non‐local bulk plasmonic effects (Figure 2f). The photothermal conversion efficiency of the thin‐film surfaces easily exceeds 100% when it involves the phenomenon of SPR or electron‐phonon coupling.^[^ ^68^, ^69^, ^70^ ^]^

After printing the carbon‐black film by the R2R gravure, the mini‐wells should be imprinted on the printed carbon‐black with good adhesion, biocompatibility, and high speed for manufacturing the PCR chips. Among many available polymer resins, PDMS was selected for imprinting the PCR chips because of its bio‐compatibility^[^ ^71^ ^]^ and ubiquity in biochips.^[^ ^72^, ^73^ ^]^ However, a long curing time (>24 hr) of PDMS precursors at room temperature impedes high‐throughput R2R manufacturing and lowers the production cost. Thus, the curing issue was mitigated by developing room‐temperature curable PDMS precursors by employing an organometal catalyst that required only 5 min to fully cure it (see methods). The formulated PDMS precursors with the organometal catalyst were used to imprint the mini‐wells on the printed carbon‐black film at the imprinting speed of 270 mm min^−1^ under ambient conditions, which can be scaled up massively in the manufacturing industry. To decide on an efficient PDMS well structure for carrying out thermal cycles of heating and cooling, the system of the PDMS‐based chip was optimized by simulating thermal rates (heating and cooling) of both cylindrical and inverted frustum of a circular cone‐shaped design with the same height of 1.4 mm and bottom radius of 1 and 0.5 mm respectively (Figure S5a, Supporting Information). We used the Lee model^[^ ^74^ ^]^ for phase change and *k – ε* turbulence for heat transfer. A detailed explanation of the simulation is discussed in Figure S5 (Supporting Information) and Note S1 (Supporting Information). Based on the simulation, the inverted frustum of a circular cone was better for attaining faster thermal heating and cooling rates because of the higher number of convection currents than in the plain cylindrical well structure. Furthermore, the simulation results are well matched the experimental data for heating and cooling of the PCR chip with the shape of an inverted frustum of a circular cone on 14 µm thickness of the carbon‐black thin film. The heating and cooling rates were 24 °C s^−1^ and −2.4 °C s^−1^, respectively, in the simulation, while experimentally, we achieved 22 °C s^−1^ and −2.6 °C s^−1^ heating and cooling rate, respectively, with 5 µL of solution in the PCR chip (Figure 2g; Figure S6, Supporting Information). Considering each cycle to be 14 s, the required 30 thermal cycles of the standard PCR can be achieved within 7 min with such low thermal latency of PDMS‐based PCR chip. For imprinting the PCR chip by the R2R imprinting system, an inexpensive flexographic printing plate (flexo plate) was used as an imprinting mold which sufficed the resolution required for a millimeter‐scaled PCR chip (Figure 2d; Figure S2c, Supporting Information). Figure S2d,e (Supporting Information) clarifies the details of the R2R imprinting process and the characteristics of the PCR chip. The production rate of PCR chips was also boosted by 7 times by employing an external heat source. The R2R gravure and imprinting technique in this work can be further optimized to quickly scale up the production rate to meet high demands in the MEDIC‐PCR‐based diagnosis during pandemics or epidemics at a low‐cost. This high‐throughput R2R manufacturing system would open a new path to cut down the current cost‐per‐PCR test.

A typical temperature profile of a single thermal cycle of the PCR is shown in Figure 2h, where the transition stage from annealing (58 °C) to denaturation (93 °C) temperatures are marked by red (≈2 s) and the cooling stage is marked in blue (≈12 s). Since the temperature profile of the PCR chip should be first calibrated to reach the desired temperature while being used with the MEDIC‐PCR, it is essential to have a constant calibration factor when manufactured by the R2R gravure imprinter. Therefore, two‐point calibration was performed to get the desired annealing and denaturation temperatures in the PCR chip. The calibration was performed using a secondary thermocouple (built‐in in the MEDIC‐PCR) for several PCR chips to get the reference temperature by which the solution temperature in the PCR chip reached the desired temperature with the MEDIC‐PCR. Figure S7, Supporting Information shows the calibration curves of 70 different PCR chips in the PCR temperature profile for four different temperature zones. The R2R‐manufactured 355 PCR chips were randomly selected to perform 40 thermal cycles with 58 °C annealing and 93 °C denaturation temperatures. The distribution of calibration reference temperatures at each cycle was plotted in Figure 2i. The distribution in the calibration temperatures showed a narrow bandwidth with peaks at 105.39 °C ± 0.34 °C (denature), and 44.56 °C ± 0.01 °C (anneal), and the data used for plotting distribution graph are presented in Figure S8 (Supporting Information). Such narrow bandwidth substantiates the consistency and reliability of R2R‐manufactured PCR chips. Henceforth, the PCR chip can perform real‐time RT‐qPCR using the MEDIC‐PCR.

### 2.2 MEDIC PCR

**Figure 3:**
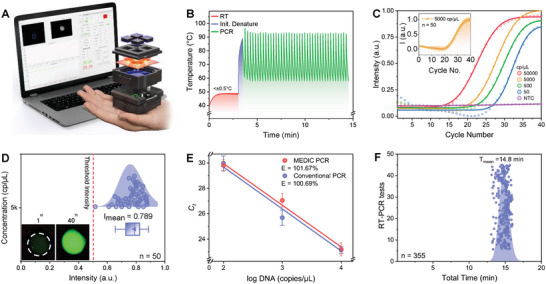
Real‐time RT‐PCR by MEDIC‐PCR. A) A compact and portable MEDIC‐PCR. The sample was loaded on the PCR chip and inserted at the top of the MEDIC‐PCR. The device was configured using the software running on a laptop. B) Rapid profile of the MEDIC‐PCR thermal cycle for the amplification of RNA using RT and PCR (14.7 min). C) Fluorescence intensity curves of amplification of N1 gene using the MEDIC‐PCR with different viral RNA concentrations. The negative control (NTC) showed no change in the intensity throughout all thermal cycles, whereas 50, 500, 5000, and 50000 copies per µL decreased the *C_t_* value. The intensity was calculated from the fluorescence images at the end of each annealing cycle by the software in real‐time. C (inset) Fluorescence intensity curves for 50 samples with 5000 copies per µL N1 gene RNA, amplified using the MEDIC‐PCR. D) Once reverse transcription and 40 thermal cycles were completed, the final normalized intensity of the fluorescence was used to determine the COVID‐19 infection. The mean intensity of the peak was 0.789, and the mean threshold intensity was 0.45, the optimum value to avoid “false positive” and “false negative.” The inset at the left bottom of the image shows the fluorescence image after the first and final thermal cycles. E) Standard‐curve plot for calculated efficiencies for conventional RT‐qPCR instrument and the MEDIC‐PCR. The efficiencies were calculated by performing three experiments with each viral concentration. F) The total time required for completing real‐time RT‐PCR by the MEDIC‐PCR was less than 15 min. We plotted the distribution curve of the entire duration for 355 experiments, and the mean duration was 14.8 min.

A fast, reliable, and miniature thermal cycler and sensitive fluorescence measurement system, along with simple hardware, software, and firmware, are indispensable for developing an inexpensive, mobile, and real‐time RT‐qPCR system with high sensitivity and accuracy.^[^ ^75^, ^76^ ^]^ To utilize the PCR chips, the MEDIC‐PCR, which consisted of a near‐IR LED (940 nm) based thermal cycler and a fluorescence reader (Dino‐lite AM4117MT‐G2FBW), was made with simple‐operation software and hardware (**Figure** **3**a) to provide high thermal stability (< ±0.5 °C fluctuation) and sensitive fluorescence readability in the PCR chips to complete the real‐time RT‐qPCR assay from a sample to the answer. A detailed explanation of the hardware design and assembly of the MEDIC‐PCR, along with simple software, is shown in Figures S9 and S10 (Supporting Information), respectively. A significant advantage of the MEDIC‐PCR is one‐step rapid and real‐time RT‐qPCR in which nasopharyngeal sample with lysis buffer was tested without filtration, RNA purification, or pre‐concentration by the rapid photothermal cycles for the PCR. Reliable and fast photothermal cycles were achieved by simply turning an LED light (940 nm) on and off to induce the photothermal conversion in the PCR chip. In general, the MEDIC‐PCR was configured to execute the real‐time RT‐qPCR within 180 s of RT (48–52 °C), 25 s of initial denaturation (93 °C), and 40 cycles of annealing (58 °C) and denaturation (93 °C) steps (Figure 3b). The temperature stability was under ±0.5 °C (Figure 3b) in the RT stage followed by 25 s of initial denaturation required for proper amplification of the target RNA. Thus, the real‐time RT‐qPCR can be completed using the MEDIC‐PCR within 15 min from a sample to the answer by keeping a small reaction volume (1 µL).

SARS‐CoV‐2 reference RNA was used as a positive control to demonstrate the direct and rapid RT‐qPCR capability of the MEDIC‐PCR with N1 gene‐probe pair (72 bp) (Table S3, Supporting Information), as recommended by the Centers for Disease Control and Prevention (CDC).^[^ ^77^ ^]^ Various concentrations (50, 500, 5000, and 50 000 copies per µL) of viral RNA of SARS‐CoV‐2 were prepared, and real‐time RT‐qPCR was performed using the MEDIC‐PCR. In Figure 3c, real‐time fluorescence intensities calculated at the end of each annealing stage are plotted, which showed low *C*_t_ for high viral concentration as the *C_t_* value changes with viral concentration. The higher the concentration lower the *C_t_* values, or vice versa. The positive amplification of the desired amplicon was further verified by utilizing a gel electrophoresis method (Figure S11, Supporting Information). In rapid real‐time RT‐qPCR, the discrete elongation stage is skipped, and the elongation transpires during the transition stage.^[^ ^78^, ^79^ ^]^ In the MEDIC‐PCR device, the reduced time for thermal cycling still provided enough enzymatic activity of the polymerase and suppressed the formation of non‐specific by‐products. Furthermore, the reliability of the MEDIC‐PCR assay was verified by conducting real‐time RT‐qPCRs using 50 samples with an RNA concentration of 5000 copies per µL. The mean characteristic amplification curve (*C_t_* = 20) with an error bar was plotted from the normalized fluorescence intensities calculated at each thermal cycle (Figure 3c inset). Since the final fluorescence intensity determines positive or negative viral infection, a proper *C*_t_ value should be set to identify the infected patient correctly. Based on the testing results in Figure 3D and setting the *C*_t_ value of 20 as “true positive,” the MEDIC‐PCR assay demonstrated 100% sensitivity for 50 samples with an RNA concentration of 5000 copies per µL. The average value of the normalized fluorescence intensity after 40 cycles was 0.789. Based on the distribution of fluorescence intensity (Figure 3d), the optimized intensity for being “true positive” must be at least 0.4 to clearly distinguish “true negative” and reduce “false positive.” The inset (Figure 3d) shows the initial and final image of the PCR solution as seen from the fluorescence camera (refer to the detailed photos in Figure S12, Supporting Information). Furthermore, the efficiency of the MEDIC‐PCR was compared with the conventional real‐time RT‐qPCR system. The standard curve was plotted to calculate the efficiency of the MEDIC‐PCR (101.67%) and the conventional RT‐qPCR (100.69%) by performing 3 PCRs per concentration (Figure 3e). Since the solution preparation for PCR took 1 min, 3 min for the reverse transcription (Figure S13, Supporting Information), and ≈10 min for 40 thermal cycles, the mean assay time was 14 min 48 s from a sample to the answer for 355 different experiments with various RNA concentrations (Figure 3f). Thus, the MEDIC‐PCR can be used to diagnose COVID‐19 in 15 min with high sensitivity and comparable efficiency to the conventional PCR system.

### 2.3 Clinical Trials

**Figure 4:**
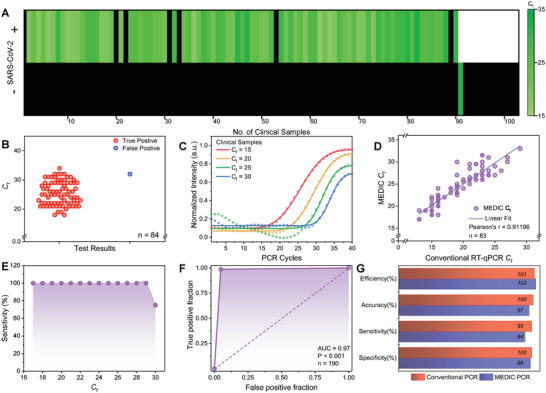
Performance of MEDIC‐PCR with COVID‐19 patients’ samples. A) *C_t_* levels of the COVID‐19 diagnosis using nasopharyngeal swabs of 89 positive and 103 negative patients amplified the N1 gene using the MEDIC‐PCR. B) 83 samples were detected as “true positive,” and 1 sample was detected as “false positive” by the MEDIC‐PCR. C) Representative amplification curves (fluorescence intensity curve) of N1 gene (SARS‐CoV‐2) amplified using the MEDIC‐PCR with *C_t_* ranging from 15 to 30 collected from patients' samples. D) *C_t_* values were determined for the samples by both the conventional RT‐qPCR instrument and the MEDIC‐PCR to calculate the Pearson's R, 0.91, which showed a strong correlation between conventional and the MEDIC‐PCR performance over *C_t_*. E) Sensitivity of the MEDIC‐PCR with different levels of *C_t_*. F) ROC curve (sensitivity vs 1‐specificity) of the MEDIC‐PCR. The AUC was 0.97 which is the accuracy of the MEDIC‐PCR. G) Summary of comparison of various parameters between the MEDIC‐PCR and the conventional RT‐qPCR system.

Clinical samples collected from the cohort of 89 COVID‐19 and 103 non‐COVID‐19 patients from Samsung Medical Center (SMC) in Seoul, Korea, preserved in universal transfer media, were tested using the MEDIC‐PCR. The *C_t_* values were acquired from the real‐time RT‐qPCR using the MEDIC‐PCR without RNA purification. The results from RT‐qPCR on 192 clinical samples (89 positive and 103 negative samples) were mapped in **Figure** **4**a, where the higher intensity of green shows low *C_t_* values. The negative samples showed no change in the fluorescence intensity at the end of the test. A total of 89 positive samples were tested with the MEDIC‐PCR; 84 samples out of 89 positive samples resulted in “true positive” (Figure 4b), while five samples resulted in “false negative.” In addition, 103 negative samples were tested, 1 out of 103 was diagnosed as a “false positive,” and 101 samples gave “true negative” results. The resulting images are grouped in an array in Figure S14 (Supporting Information), and the results are summarized in Table S4 (Supporting Information). The clinical samples were divided concerning *C_t_* values calculated by the conventional PCR system, which was proportional to the viral concentration. The MEDIC‐PCR was used to amplify the clinical samples classified by *C_t_* ranging from 15 to 30 and plotted representative fluorescence characteristic curves for each *C_t_* (Figure 4c). The samples with high *C_t_* values represented the presence of low viral concentration. The clinical performance of the MEDIC‐PCR was compared with the performance of the conventional RT‐qPCR by plotting *C_t_* values. The Pearson r coefficient was 0.91, which proved the existence of a correlation between the conventional RT‐qPCR and the MEDIC‐PCR (Figure 4d). The sensitivity for each *C_t_* was measured, and near linear sensitivity up to *C_t_* value of 29 was observed using the MEDIC‐PCR (Figure 4e). While the MEDIC‐PCR is capable of sensing as low as *C*_t_ value of 32, the tradeoff was made with sensitivity (Figure S15, Supporting Information). Receiver Operating Characteristic (ROC) curve was also obtained from the clinical results (Figure 4f). The accuracy of diagnosis was measured by the area under the ROC curve (AUC) which is a graphical representation of the “false positive” rate (x‐axis) and the “true positive” rate (y‐axis) for each possible cut‐off point of the diagnostic test.^[^ ^80^ ^]^ When the AUC is closer to 1, the more accurate the diagnostic test is. We achieved AUC of 0.97, a sensitivity of 94%, and a specificity of 98% with the MEDIC‐PCR. Based on the clinical test with the MEDIC‐PCR, we summarized the characteristics of the MEDIC‐PCR to diagnose COVID‐19 with a conventional RT‐qPCR system (Figure 4g). Efficiency, accuracy, sensitivity, and specificity were comparable with the conventional one. At the same time, the testing time from a sample to the answer was greatly reduced to 1/24^th^ of the conventional one.

## 3 Discussion

MEDIC‐PCR, a palm‐size, portable, and rapid real‐time RT‐qPCR device, has been demonstrated. The MEDIC‐PCR adopted two novel methods to dramatically reduce the assay time from 4‐6 h^[^ ^81^, ^82^ ^]^ to 15 min. First, the nucleic acid extraction step was omitted by utilizing a direct RT‐qPCR. Only chemical‐based lysis was carried out quickly by adding surfactants or molecules with an amine functional group. Since these chemicals did not inhibit the activities of reverse transcriptase and polymerase,^[^ ^83^, ^84^ ^]^ viral RNA was reverse transcribed to complementary DNA and then well amplified in a one‐pot. Second, the time to carry out the reverse transcription and 40 thermal cycles, annealing, and denaturation cycles for the amplification was reduced to 13 min by replacing a conventional thermal cycler with a photothermal cycler. In general, efficient and ultrafast photonic PCR by plasmonic metal structures using gold, silver, or aluminum has been reported.^[^ ^85^, ^86^ ^]^ However, to develop a low‐cost POC device, carbon‐black was selected to replace the novel metals to attain “light‐to‐heat” conversion in the photothermal cycler. Thus, a PCR chip was designed to replace a conventional thermocycler for executing heating and cooling cycles in the PCR by simply “ON” and “OFF” a near‐IR LED light on the printed carbon‐black thin film. The near‐IR LED light with a 940 nm wavelength efficiently converted light into heat via electron‐phonon and phonon‐phonon couplings in the printed carbon‐black thin film in the PCR chips. Carbon‐graphene‐based ink is significantly cheaper than other materials used for plasmonic heating, like Au and Ag. Although PDMS is slightly expensive, with R2R gravure printing and imprinting, the plasmonic mini‐wells can be fabricated at room temperature with no waste of PDMS as a typical high throughput additive manufacturing method. Therefore, the photothermal mini‐well production cost was extremely low, and the estimated cost is 1 cent per mini‐well. Since each test utilized just 1 µL of total PCR solution, the reagent cost per test is estimated to be $1.25, and including 1 cent for the mini‐well, each test costs ≈$1.26. Since the carbon‐black is inexpensive and robust to formulate as ink, we could employ the R2R gravure printing method to print the carbon‐black thin film and consequently in‐line imprint the PDMS‐based mini‐wells to hold the PCR solution. While printing carbon‐black thin films, micro‐sized pores could be generated due to improper ink transfer and would degrade the signal‐to‐noise ratio (SNR) while measuring the fluorescence signal due to interference from leaked light. The existence of micropores also resulted in high fluctuations of photothermal conversion and eventually affected the temperature stability of the film. In consequence, the micropores were removed by printing multiple layers of carbon‐black. The thickness of 14 µm printed carbon‐black thin film sufficed to provide a reliable and stable photothermal cycle without light leakage during fluorescence measurement. Thus, the R2R gravure printing and imprinting methods were integrated as a large‐scale additive R2R manufacturing platform for mass‐producing the PCR chips without any hazardous byproduct. Although the current R2R manufacturing speed for the PCR chips array is 15 cm min^−1^, the imprinting speed can be raised to 10 times faster by reformulating PDMS precursors with Pt catalyst or UV curable polymer resins. Furthermore, this R2R manufacturing platform can be expanded to manufacture microfluidic digital PCR chips by changing the current imprinting mold with millimeter‐sized wells to the high‐resolution one with micrometer‐sized microfluidic chips.

Using clinical samples (nasopharyngeal swabs) from the cohort of 192 patients, the MEDIC‐PCR proved its potential as a personalized, accurate, and convenient POC device to diagnose SARS‐CoV‐2 within 15 min, starting from a sample to the answer by monitoring the fluorescence intensity in real‐time. Since the fluorescent intensity data was collected by capturing the fluorescence images at the end of each annealing cycle and calculating the average intensity of the fluorescence area, errors would be generated due to the artifacts such as the formation of bubbles in the PCR solution. The bubbles were induced due to thermal agitation of the trapped air in the PDMS micro‐pores and the momentary high temperature at the bottom of the well. We observed that, adding polyethylene glycol (5 wt.%) in the PCR reagent resolved the bubble formation caused by transient high temperatures. On the other hand, the bubble formation due to trapped air in the porous PDMS was determined by coating the surface of the PCR chip with hydrophilic material.^[^ ^87^ ^]^

In our clinical tests, we used N1 gene as a target for the RT‐qPCR tests using both conventional RT‐qPCR (the Applied Biosystems 7500 Real‐time PCR Instrument system of Thermo Fisher Scientific) with 20 µL sample volume after traditional sample preparations and MEDIC‐qPCR with 1 µL sample volume. We respectively achieved *C_t_* values of 29 and 33 for the same specimen. The equivalent copy numbers in the reaction volume of 1 µL (MEDIC) and 20 µL (SMC) were calculated to be 19 and 290 copy numbers based on the RT‐qPCR standard curve of SARS‐CoV‐2 (N1 gene) achieved with 20 µL of reaction volume by US CDC.^[^ ^77^ ^]^ Thus, in our clinical tests, the limit of detection (LoD) for the MEDIC‐qPCR was 19 copies per reaction (1 µL). While analyzing the result of the clinical tests, the *C_t_* values of the MEDIC‐qPCR were 4.99 higher on average than the conventional RT‐qPCR's *C_t_* values (Table S6, Supporting Information) as the MEDIC‐qPCR used direct sample preparation without RNA extraction and a small sample volume (1 µL) while conventional RT‐qPCR test used RNA extraction and 20 µL of reaction volume. Also, in the tests conducted in the laboratory using the reference material, we achieved the LoD up to 40 copies per mL, equivalent to 1 copy per reaction (25 µL) (Figure S11c, Supporting Information), which is analogous to 7 days after the SARS‐CoV‐2 infection.^[^ ^88^, ^89^ ^]^

Since the MEDIC RT‐qPCR was designed for simultaneous PCR tests (4x), negative control (NC) and positive control (PC) can be realized in future commercial MEDIC RT‐qPCR devices. However, our current system uses a commercially available fluorescence detection camera with a narrow field of view that can focus only on a single mini‐well and provide adequate excitation light intensity for a single mini‐well. Therefore, NC and PC can be detected with the endpoint method at this moment. However, upgrading the fluorescence detection camera with a wider field of view allows four samples to be tested using four different mini‐wells in the MEDIC simultaneously with sufficient excitation light intensity for real‐time quantitative analysis with NC and PC samples included. Furthermore, MEDIC‐PCR can be upgraded from a wired connection to the personal computer (PC)/laptop to a wireless device that can transmit the obtained quantitative real‐time PCR data to the hospitals wirelessly so that the illness severity and comorbidity can be remotely diagnosed and quickly treat the infected persons at the primary or urgent care levels.

## 4 Conclusion

The advantages of MEDIC‐PCR over conventional real‐time RT‐qPCR are low‐cost, mobile, compact size, and fast assay in completing sample‐to‐answer within 15 min while keeping similar accuracy, efficiency, specificity, and sensitivity. In addition, the PCR chips, the core of MEDIC‐PCR, have a practical advantage in manufacturing. By utilizing inexpensive ubiquitous materials like carbon black, PDMS, and PET and taking advantage of the R2R manufacturing system, the MEDIC‐PCR can be a prospect POC platform that has a sustainable manufacturing method for infectious disease diagnostics. The MEDIC‐PCR can further reduce the time for the sample to the answer by employing highly thermally conductive materials such as TiO_2_, Al_2_O_3,_ and MgO in the PDMS wells.^[^ ^90^, ^91^ ^]^ By increasing the thermal conductivity of the PDMS wells, 40 thermal cycles could be completed in less than 3 min. Furthermore, since the fluorescence intensity can be enhanced by adding TiO_2_ in the PDMS well,^[^ ^92^ ^]^ the sensitivity can be improved more than three times.^[^ ^93^, ^94^ ^]^Moreover, the sensitivity can also be improved to the comparable value as conventional PCR by increasing the volume to 10‐20 μL. Having tested with the COVID‐19 N1 gene, the MEDIC‐PCR demonstrated 102% efficiency, 97% accuracy, 94% sensitivity, and 98% specificity. Furthermore, the MEDIC‐PCR showed excellent agreement with the conventional RT‐qPCR with a Pearson coefficient of 0.91, calculated from the clinical tests of 192 patient samples. Also, since a self‐adhesive cover film can replace a mineral oil used to cover the PCR solution, and a smartphone can easily replace the laptop/PC in this work, the MEDIC‐PCR can provide a promising platform for effectively preventing future pandemics by helping an individual to make quick and accurate self‐diagnosis with instant results. Furthermore, since the MEDIC‐PCR can provide quantitative real‐time data and it can be made to transmit the attained data to hospitals wirelessly, the illness severity and comorbidity can be remotely diagnosed and quickly treated the infected persons at the primary or urgent care levels.

## 5 Experimental Section

### R2R Gravure Print and Imprint

Five units of a roll‐to‐roll printing system (Figure S2, Supporting Information) were used, namely the unwinder, first gravure unit, second gravure unit, imprinting unit, and finally rewinder unit from left to right. Rolls with 270 mm width were used to print and imprint the carbon‐black thin films and PDMS‐based PCR chips. Carbon‐black ink with 1000 *C_p_* viscosity was used, prepared by mixing the solvent of diethylene glycol mono butyl ether (Sigma Aldrich) and carbon‐black paste (Dozentech Korea) using a mechanical stirrer at room temperature for 2 h. The flexographic plate‐based mold was first designed using AutoCAD software. The designed flexographic plate was fabricated by Hyunjin Flexo in Korea.

### MEDIC‐PCR Thermal Cycler

The MEDIC‐PCR thermal cycler was designed with four near‐IR light emitting diodes (LEDs) (LZ4‐00R708) operated at 3.75 W optical power, which could allow the operation of four simultaneous PCRs. A medical application‐approved power supply TPP 30A‐J (30 W max) was used with a 3D printed casing. K‐type thermocouple (5SRTC‐TT‐(K)−40‐36) was fixed just above the LED with a gap of 500 µm for feedback temperature in the closed‐loop control system. MCP9600 was used to measure the hot and cold junction of the thermocouple at 20 Hz of a sampling rate which also performed the cold junction compensation. Software‐based closed loop Proportional Integral Derivative (PID) control system was implemented to reach all the RT‐qPCR thermal states by utilizing Pulse Width Modulation (PWM) of 490 Hz to control the intensity of the LEDs. The fluorescence camera (AM4117MT‐G2FBW) with an inbuilt 465 nm excitation filter and 510 – 545 nm emission filters was purchased from Dino‐lite Korea and used without modification. The overall MEDIC‐PCR device was 3D printed with a footprint of 62 × 62 × 48 mm, excluding the camera. The MEDIC‐PCR was designed with matte‐finished black plastics, sustainable at 300 °C. The estimated cost of the MEDIC‐PCR thermal cycler is shown in Table S5 (Supporting Information).

### MEDIC‐PCR Software

The software was developed using MATLAB and runs on any computer including laptops. The software configures the MEDIC‐PCR device every time to run a specific diagnostic. The excitation LEDs were turned on at 4 °C (adjustable) before reaching the annealing temperature to capture the fluorescence images. It was found that exciting the fluorescence only at the instance of capturing the image could result in a misleading calculation of the fluorescence intensity due to the improper lighting duration and latency in capturing an image with the camera's shutter speed. Therefore, the program was developed to excite fluorescence a few degrees before the annealing point to adjust the misleading calculation. In the whole experiment, all configuration parameters of the camera were fixed to get a consistent result. The matte black color of the MEDIC‐PCR body improved the signal‐to‐noise ratio while capturing the fluorescence images by completely shielding external lights and reducing the probable reflection.

### PDMS Synthesis

Vinyl‐terminated PDMS, co‐methylhydrosiloxane copolymer, platinum (0)−1,3‐divinyl‐1,1,3,3‐tetramethyldisiloxane complex solution, and dimethyl maleate were all purchased from Sigma Aldrich and used without further purification or treatment to imprint the PDMS. Dimethyl maleate was used as an inhibitor to properly control the curing rate caused by the platinum (Pt) catalyst, and xylene was used as a dilution agent. A double‐jacket reactor chamber was used to uniformly mix the vinyl‐terminated PDMS and co‐methylhydrosiloxane copolymer in a ratio of 5:1. The copolymer was added and stirred at a constant speed of 100 RPM for 10 min in the vacuum to prevent bubble formation, during the entire time the internal temperature of PDMS was maintained at −5 °C to prevent a pre‐curing reaction. Next, 0.01 v/v% of catalyst was injected to prepare the final room temperature curable PDMS.

### MEDIC PCR Mixture Preparation

The *λ*‐DNA was purchased from Roche Applied Science. KAPA2G Fast DNA Polymerase (2X) containing 1.5 mm MgCl_2_ and 0.2 mm dNTP mixed at 1X was also purchased from Roche Applied Science for rapid PCR. The forward and reverse primer was purchased from Cosmogenetech. The PCR sample solution with the *λ*‐DNA target amplicon size was 55 and 100 bp including 1X KAPA2G polymerase, 0.8 µm of each primer in 1 µL of PCR solution. Furthermore, the SARS‐CoV‐2 RNA reference material was purchased from KRISS. The Arcis One‐Step Viral Sample Preparation Kit for lysis was purchased from 2NBIO, Inc., and One Step PrimeScript RT‐qPCR Mix (2X) was purchased from TAKARA. The forward primer, reverse primer, and TaqMan probe was purchased from IDT. The RT‐qPCR sample solution with the reference SARS‐CoV‐2 RNA target amplicon (size of 72 bp N1 gene) including 2X of One Step Primescript RT‐qPCR Mix, 0.8 µm of each primer, 0.04 µm of the probe, and 5 wt.% of PEG were included in 1 µL of total volume.

### Gel Electrophoresis

Products of RT‐PCR were separated by electrophoresis on a dry precast 4% agarose gel (Invitrogen) containing 1X SYBR safe (Invitrogen) as a staining dye for 15 min. The lengths of the products were determined relative to a 50 bp DNA Ladder (Invitrogen). Images were taken using an all‐in‐one E‐Gel^TM^ power snap electrophoresis system (Invitrogen).

### Preparation of PCR Solution for a Clinical Study

Clinical samples were tested at Samsung Medical Center (SMC) in Seoul, Korea using a PowerCheck SARS‐CoV‐2 Real‐time PCR kit (Kogene Biotech) following the manufacturer's instructions. An automatic nucleic acid extractor was used to extract nucleic acids for standard qPCR tests. The nucleic acid was automatically extracted from 200 µL of a raw specimen using the Libex nucleic acid extractor of Xi'an Tianlong Technology CO., Ltd. The extractor used 7 steps, starting with lysis, combining, washing 1, 2, 3, elution, and release, and required ≈37 min in total. For conventional PCR tests, the Applied Biosystems 7500 Real‐time PCR Instrument system (Thermo Fisher Scientific) was used with the following cycling conditions: 50 °C for 30 min, 95 °C for 10 min, and 40 cycles of 95 °C for 15 s, and 60 °C for 1 min. RT‐qPCR was conducted with the MEDIC‐PCR device with 1 µL of PCR solution and 4 µL of mineral oil. For the PCR test using MEDIC‐PCR, samples were prepared as follows: First, 2 µL was collected from a universal transport medium (UTM) and then mixed with 2 µL of lysis buffer. Second, 2 µl of lysed was added to mix from the previous step into 23 µl master mix, and finally, 1 µL of the prepared solution was added into the mini‐well for testing. The device was configured using the computer software and was set to 50 °C for reverse transcription, 93 °C for denaturation, 58 °C for annealing, and 40 thermal cycles. The *C*_*t*_ value was calculated from the achieved fluorescence intensity graph.

### Clinical Study

The nasopharyngeal and oropharyngeal samples were collected and provided by SMC. All the clinical tests were performed at the facility of the SMC. The tests were conducted with 89 positive and 103 negative COVID‐19 samples with the N1 gene as a target gene. The *C_t_* values of these samples were pre‐tested by the Applied Biosystems 7500 Real‐time PCR Instrument system (Thermo Fisher Scientific) at SMC (Table S6, Supporting Information). This study received ethical approval from the Institutional Review Board (IRB) at SMC with the following IRB number: IRB‐2021‐01‐036.

## Conflict of Interest

The authors declare no conflict of interest.

## Supporting information

Supporting Information

## Data Availability

The data that support the findings of this study are available from the coresponding author upon reasonable request.
