# Supplementary material for: Mobile Efficient Diagnostics of Infectious Diseases via On‐Chip RT‐qPCR: MEDIC‐PCR
Source: Adv Sci (Weinh). 2023 Aug 16;10(28):2302072. doi: 10.1002/advs.202302072 (PMC10558658; doi:10.1002/advs.202302072)
Supplement: Supplementary file 1 — Supporting Information [file ADVS-10-2302072-s001.pdf]

ADVANCED SCIENCE
Open Access

# Supporting Information

for *Adv. Sci.*, DOI 10.1002/advs.202302072

Mobile Efficient Diagnostics of Infectious Diseases via On-Chip RT-qPCR: MEDIC-PCR

*Kiran Shrestha, Seongryeong Kim, Jiyeon Han, Gabriela Morales Florez, Han Truong, Trung Hoang, Sajjan Parajuli, Tiara AM, Beomsoo Kim, Younsu Jung, Abdurhaman Teyib Abafogi, Yugyeong Lee, Seung Hyun Song, Jinkee Lee, Sungsu Park, Minhee Kang, Hee Jae Huh, Gyoujin Cho* and Luke P. Lee**

# Supporting Information

## Mobile efficient diagnostics of infectious diseases via on-chip RT-qPCR: MEDIC-PCR

*Kiran Shrestha, Seongryeong Kim, Jiyeon Han, Gabriela Morales Florez, Han Truong, Trung Hoang, Sajjan Parajuli, Tiara A.M, Beomsoo Kim, Younsu Jung, Abdurhaman Teyib Abafogi, Yugyeong Lee, Seung Hyun Song, Jinkee Lee, Sungsu Park, Minhee Kang, Hee Jae Huh, Gyoujin Cho*, and Luke P. Lee**

**Table S1.** The equivalent optical power of different wavelength LED to its electrical power.

| Wavelength (nm) | Electrical Power (mW) | Optical Power (mW) |
|---|---|---|
| 465 | 533 | 170 |
| 530 | 2450 | 170 |
| 660 | 485 | 168 |
| 940 | 652 | 170 |

**Table S2.** The minimum and maximum temperature and response time to calculate the photothermal conversion efficiency of carbon-graphene thin film.

| **Thickness (μm)** | **$T_{min}$ (ºC)** | **$T_{max}$ (ºC)** | **△T (ºC)** | **Time (sec)** | ***η* (%)** |
|---|---|---|---|---|---|
| 4 | 27.44 | 117.62 | 90.18 | 2.048 | 93.9 |
| 7 | 27.44 | 117.42 | 89.98 | 1.994 | 96.2 |
| 14 | 27.44 | 118.56 | 91.12 | 1.997 | 97.3 |
| 21 | 27.44 | 117.12 | 89.68 | 2.714 | 70.5 |

**Table S3.** PCR and RT-PCR primers and probes for Lambda DNA, Human coronavirus 229E (COV 229E), and SARS-CoV-2 (N1 gene) detection as recommended by the CDC.

| Target | Specification | Oligonucleotide sequence (5'à 3') | Amplicon size (bp) |
|---|---|---|---|
| λ-DNA | Forward primer | ATG CTG AAG TGA TGG CAG AGC GGA AA | 55 |
| | Reverse primer | CGG TCA GGA ACG GGC GCT GAA TAA | |
| | Forward primer | CAT CGT CTG CCT GTC ATG GGC TGT TAA T | 100 |
| | Reverse primer | TCG CCA GCT TCA GTT CTC TGG CAT TT | |
| COV 229E | Forward primer | TTT CTT ATT TCT CAA CTA ACG ACT TGT ACG | 117 |
| | Reverse primer | CAC AAA ACA AGT ATA CAC ATT CAC CGT TAT ATA | |
| SARS-CoV-2 | N1 gene forward primer | GAC CCC AAA ATC AGC GAA AT | 72 |
| | N1 gene reverse primer | TCT GGT TAC TGC CAG TTG AAT CTG | |
| | N1 gene probe | ACC CCG CAT TAC GTT TGG TGG ACC | |

**Table S4** The decision matrix shows responses to a binary diagnostic test, according to the status of each sample.

| Category of test result | Disease status (gold standard) | | |
|---|---|---|---|
| | Positive (D+) | Negative (D-) | Total |
| Positive (T+) | 77 (True positive) | 3 (False positive) | 80 (test positives) |
| Negative (T-) | 12 (False negative) | 100 (True negative) | 112 (test negatives) |
| Total | 89 (diseased) | 103 (non-diseased) | 192 (sample size) |

Sensitivity = true positive fraction = (TP/(TP+FN))
Specificity = true negative fraction = (TN/(TN+FP))
Accuracy = ((TP+TN)/(TP+FP+FN+TN))
False negative rate = (FN/(FN+TP))
False positive rate = (FP/(FP+TN))

**Table S5.** The expected cost for MEDIC device.

| Components | Cost (USD) | Quantity (pcs) | Source | Qty per MEDIC |
|---|---|---|---|---|
| PCB | 360 | 1000 | pcbway.com | 1 |
| ATMEGA328PB-MU | 1428 | 1000 | digikey.com | 1 |
| MCP9600-E/MX | 26680 | 4000 | digikey.com | 4 |
| Passive electronic components (R, C, Ferrite) | 75.01 | 61000 | digikey.com | 61 |
| MMBT5551LT1G | 159.54 | 4000 | digikey.com | 4 |
| IRLML6402TRPBF | 156.22 | 4000 | digikey.com | 4 |
| FT230XQ-R | 1712.50 | 1000 | digikey.com | 1 |
| USB connector | 289.88 | 1000 | digikey.com | 1 |
| Power Connector | 368.30 | 1000 | digikey.com | 1 |
| | | | | |
| Camera AM4117MT-CFVW | 1089000 | 1000 | dinolite.us | 1 |
| Power supply TPP 30-112A-J | 34037 | 1000 | tracopower.com | 1 |
| | | | | |
| 3D printed case | 422.50 | 50 | pcbway.com | 1 |
| | | | | |
| Thermocouple CHAL-003-36 | 94.44 | 4572 mm | omega.com | 400 mm |
| | | | | |
| | | | | |
| Item | Quantity | Cost per Unit ($) | | |
| Thermocycler | 1 | 81.95 | | |
| Fluorescence detection camera | 1 | 1089 | | |
| Total cost per MEDIC | | 1170.95 | | |

**Table S6.** Table of $C_t$ values of standard qPCR and MEDIC qPCR.

| | Conventional PCR $C_t$ | MEDIC PCR $C_t$ | $C_t$ diff. | | Conventional PCR $C_t$ | MEDIC PCR $C_t$ | $C_t$ diff. | | Conventional PCR $C_t$ | MEDIC PCR $C_t$ | $C_t$ diff. |
|---|---|---|---|---|---|---|---|---|---|---|---|
| 1 | 15 | 19 | 4 | 29 | 16 | 22.5 | 6.5 | 57 | 16 | 22 | 6 |
| 2 | 18 | 24 | 6 | 30 | 21 | 26 | 5 | 58 | 13 | 17 | 4 |
| 3 | 16 | 18 | 2 | 31 | 15 | 18 | 3 | 59 | 15 | 19 | 4 |
| 4 | 19 | 23 | 4 | 32 | 16 | 22.5 | 6.5 | 60 | 25 | 27 | 2 |
| 5 | 15 | 18 | 3 | 33 | 22 | 29.5 | 7.5 | 61 | 18 | 23 | 5 |
| 6 | 16 | 20 | 4 | 34 | 21 | 26 | 5 | 62 | 16 | 21.5 | 5.5 |
| 7 | 23 | 30 | 7 | 35 | 16 | 21 | 5 | 63 | 19 | 24.5 | 5.5 |
| 8 | 16 | 21 | 5 | 36 | 23 | 28 | 5 | 64 | 15 | 19 | 4 |
| 9 | 15 | 18 | 3 | 37 | 23 | 27 | 4 | 65 | 16 | 20 | 4 |
| 10 | 13 | 19 | 6 | 38 | 15 | 22 | 7 | 66 | 22 | 27 | 5 |
| 11 | 15 | 20 | 5 | 39 | 16 | 21 | 5 | 67 | 16 | 20 | 4 |
| 12 | 20 | 24 | 4 | 40 | 27 | 31 | 4 | 68 | 21 | 29.5 | 8.5 |
| 13 | 23 | 27 | 4 | 41 | 19 | 27 | 8 | 69 | 21 | 26 | 5 |
| 14 | 26 | 28 | 2 | 42 | 24 | 31 | 7 | 70 | 23 | 29 | 6 |
| 15 | 24 | 27 | 3 | 43 | 19 | 23 | 4 | 71 | 22 | 26 | 4 |
| 16 | 14 | 19 | 5 | 44 | 18 | 24 | 6 | 72 | 25 | 29 | 4 |
| 17 | 15 | 19 | 4 | 45 | 16 | 21 | 5 | 73 | 22 | 29.5 | 7.5 |
| 18 | 19 | 22.5 | 3.5 | 46 | 15 | 21.5 | 6.5 | 74 | 23 | 31.5 | 8.5 |
| 19 | 15 | 20.5 | 5.5 | 47 | 24 | 27 | 3 | 75 | 23 | 26 | 3 |
| 20 | 20 | 25.5 | 5.5 | 48 | 19 | 30 | 11 | 76 | 29 | 33 | 4 |
| 21 | 21 | 26 | 5 | 49 | 17 | 22 | 5 | 77 | 22 | 26 | 4 |
| 22 | 19 | 24 | 5 | 50 | 24 | 28 | 4 | 78 | 21 | 26 | 5 |
| 23 | 20 | 26 | 6 | 51 | 16 | 21 | 5 | 79 | 22 | 27 | 5 |
| 24 | 23 | 26 | 3 | 52 | 19 | 25 | 6 | 80 | 21 | 25 | 4 |
| 25 | 16 | 24 | 8 | 53 | 14 | 19 | 5 | 81 | 21 | 26 | 5 |
| 26 | 19 | 23 | 4 | 54 | 21 | 26.5 | 5.5 | 82 | 23 | 31.5 | 8.5 |
| 27 | 16 | 21 | 5 | 55 | 24 | 27 | 3 | 83 | 27 | 30 | 3 |
| 28 | 21 | 28.5 | 7.5 | 56 | 13 | 17 | 4 | | | | |

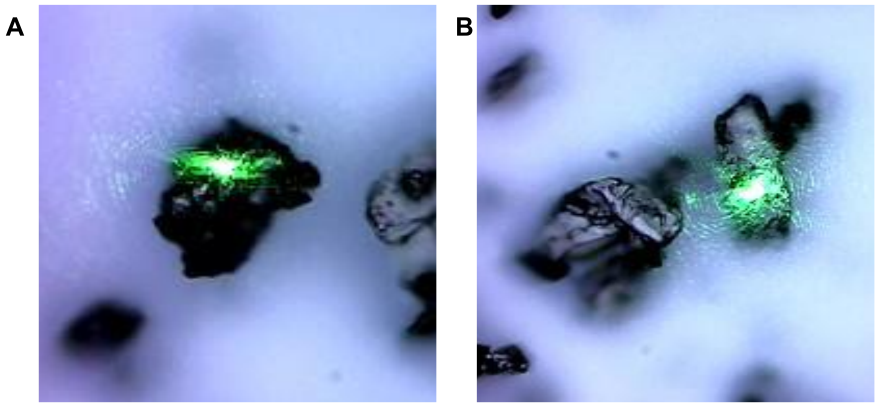


**Figure S1. Images of the sample used for the Raman spectroscopy measurement in main Figure 2C** (**A**) non-shiny area is the amorphous carbon content (**B**) graphene enriched area in the sample (shiny).

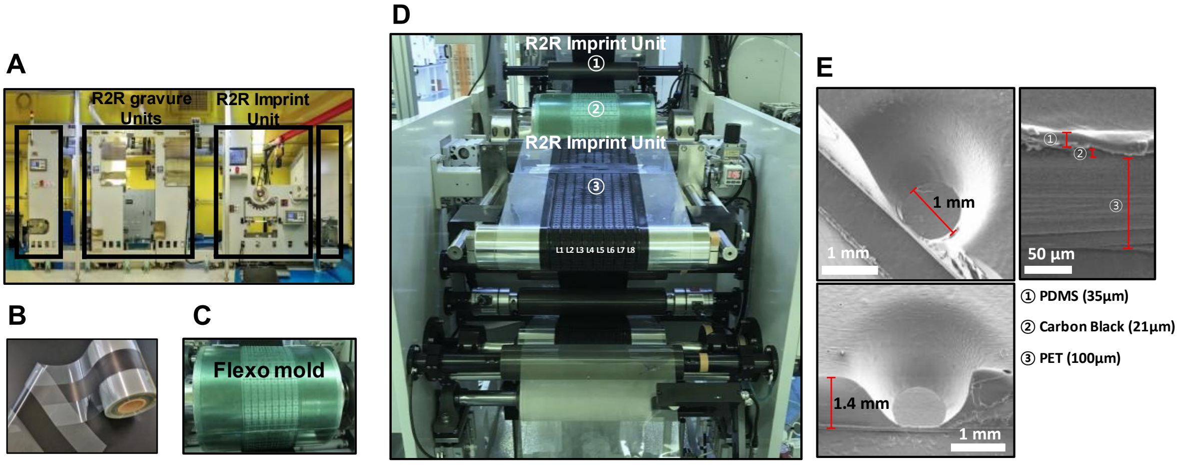


**Figure S2. R2R gravure and R2R Imprinter units.** (**A**) Five units of R2R system: unwinder unit, two units of gravure printing, imprinting unit, and unwinder unit from left to right. The R2R gravure was used to print the carbon-black on the PET substrate. Carbon-black thin films with various thicknesses (14 to 21 μm) were prepared by printing multiple times which also helped to eradicate the micro-sized holes on the film. (**B**) R2R gravure printed carbon-black thin films with 120 mm width on the PET film (width 250 mm). (**C**) The PCR chips were imprinted at the imprinting unit, and the impression roll was wrapped around by a flexographic plate mold with frustum of a circular cone pattern of 1.4 mm height. The total length of the flexographic plate mold was 816 mm and contained 472 PCR chips. The flexographic plate mold was also additionally coated with parylene-C to improve the contact angle of PDMS for enhancing the demolding process. (**D**) The PCR chips are labeled as L1 to L8 from left to right for the characterization. Marks 1, 2, and 3 highlight the incoming carbon-black thin film, imprinting roll with the pattern, and imprinted pattern respectively. (**E**) Cross-sectional, and tilted SEM images of the imprinted PCR chip. Thicknesses of PET, carbon-black, and PDMS were respectively 100, 21, and 35 μm.

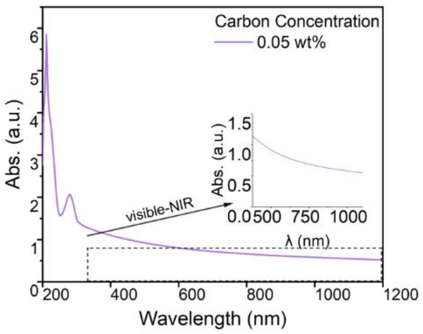


**Figure S3. UV-Vis near-IR spectroscopy.** UV-Vis near-IR spectroscopy was used to observe the absorption intensity of carbon-black. Although the resonance peaks were observed in the UV region, near-IR light was chosen due to possible effects on DNA by UV, and to avoid any interference by fluorescence reporters.

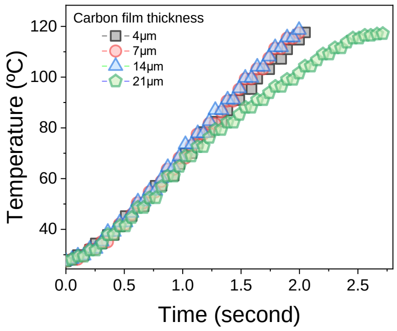


**Figure S4. Photothermal conversion efficiency of carbon black with different thicknesses.** Temperature profiles of the printed carbon-black thin films were measured with thicknesses of 4, 7, 14, and 21 μm, respectively, while exposed with 940 nm LED (3.75 W) for 2 s. Using the Equation S1, the photothermal conversion efficiency was calculated with the parameters listed below. Thus, the highest efficiency was calculated at 14 μm thickness. The temperature change of the film and response time is prepared and shown in Table S3.

$$\eta = \frac{Q}{Q_{total}} = \frac{mC_p\Delta T}{ISt} \quad \text{(S1)}$$

*Cp* = specific heat capacity = 0.72 J/g ºC
*m* = mass = 4mg
$\triangle T$ = temperature change of the film
*I* = light intensity = 0.3 W/$mm^2$
*S* = effective area = 0.0045 $mm^2$
*t* = response time

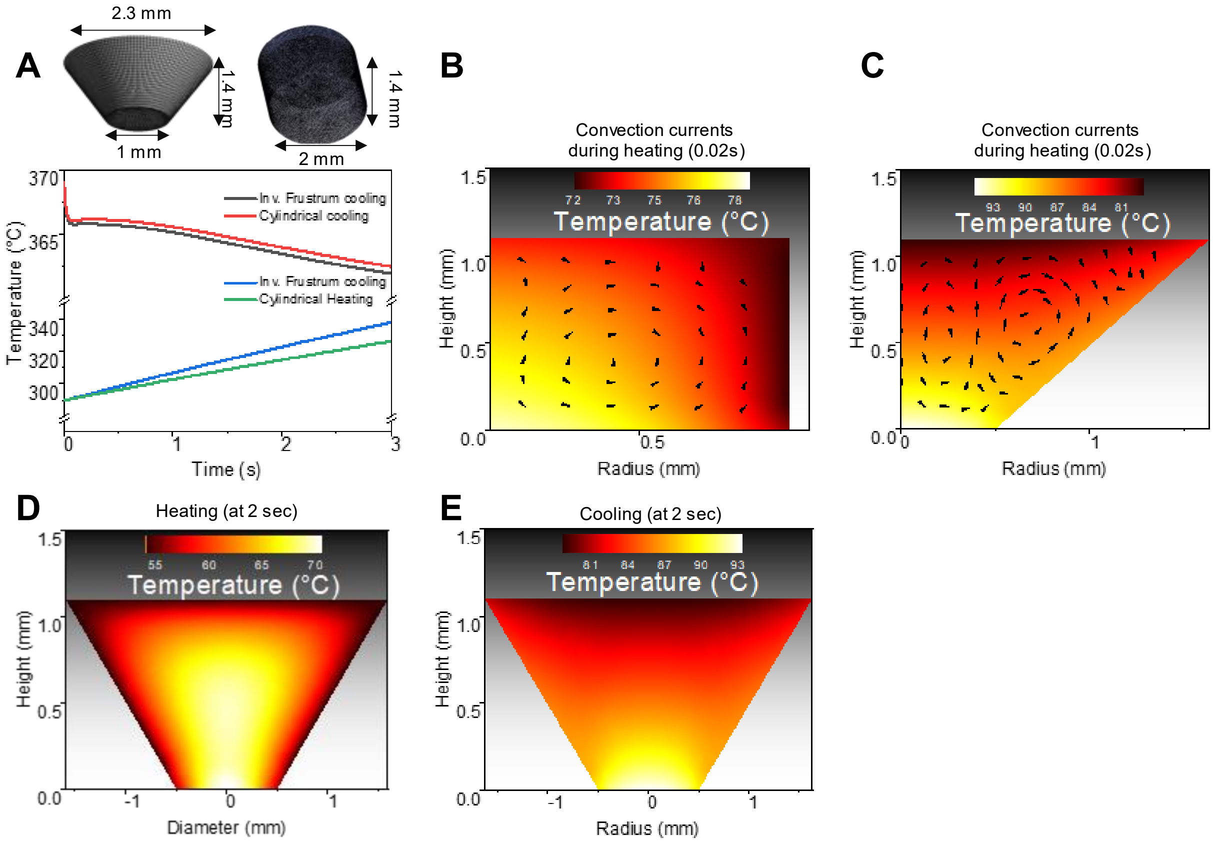


**Figure S5. Analysis of the thermal performance of PDMS chip (mini-well) using simulation.** (**A**) Heating and cooling profile of inverted frustum and cylindrical shaped PCR chip. (**B-C**) 2-D temperature contours of cylindrical and inverted frustum-shaped PCR chip, showing the convection currents during heating (indicated by arrows). The higher convection currents in an inverted frustum-shaped PCR chip proved to have higher thermal performance than in a cylindrical one. (**D-E**) 2-D temperature contours of inverted frustum-shaped PCR chip, showing the temperature profile of heating and cooling after 2 and 12 s, respectively.

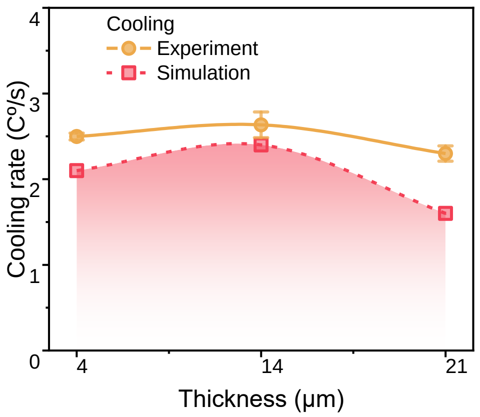


**Figure S6. Experimental and simulation results of the PCR chip during the cooling cycle**.

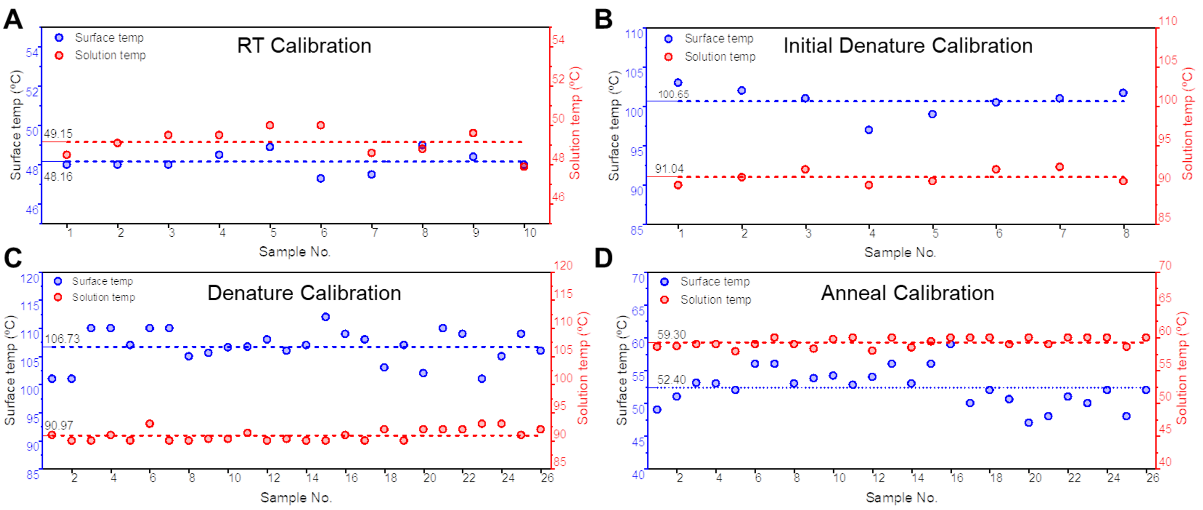


**Figure S7. Calibration of the PCR chips to control temperatures in the PCR solution.** (**A-D**) The calibration curves of PCR chips of the MEDIC for different temperature levels required for RT-qPCR. The blue line represents the calibration reference temperature, while the red line represents the temperature of the PCR solution inside the PCR chip.

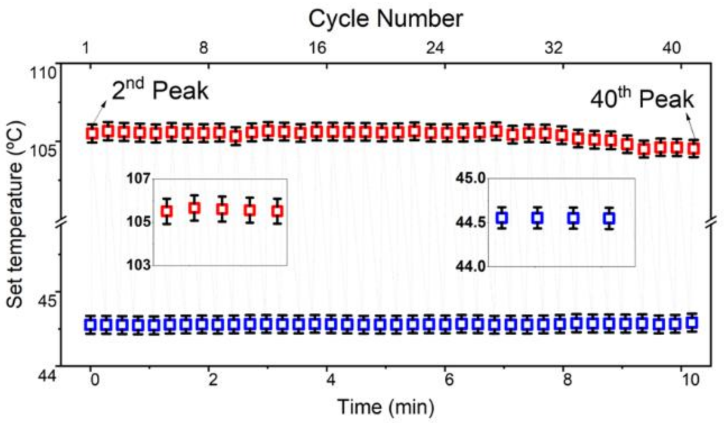


**Figure S8. Annealing and denaturing temperature of the 355 PCR chips.** Denaturing and annealing temperatures of 355 experiments were plotted with error bars.

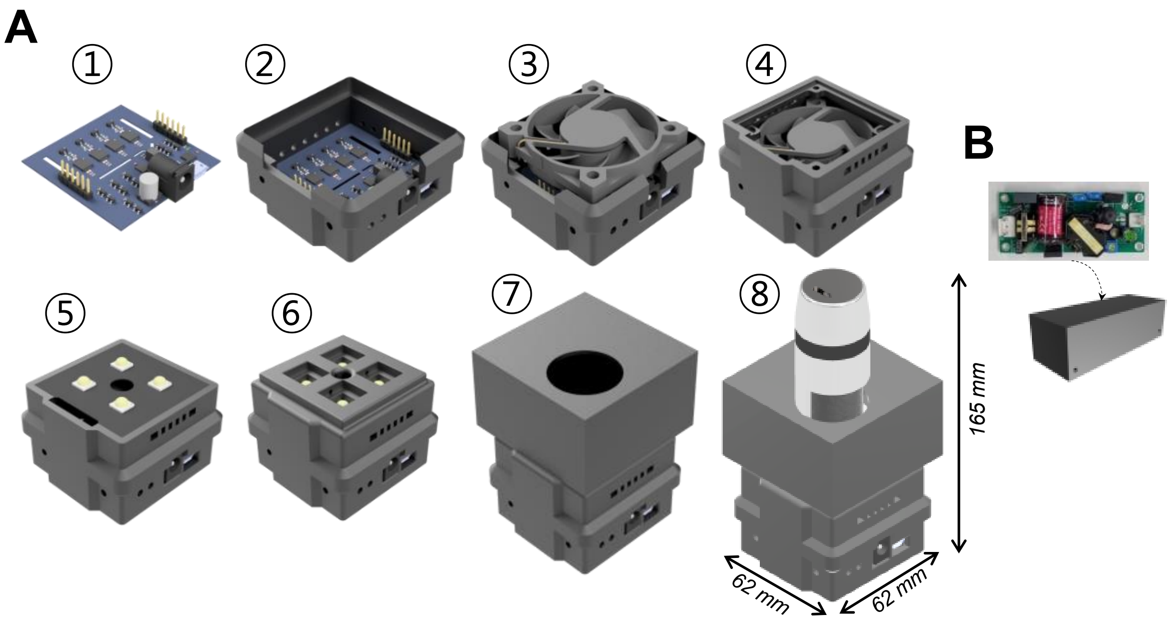


**Figure S9 Detailed information on the MEDIC device's assembly and parts.** (**A**) The MEDIC device was made with 3D-printed plastic that withstands a temperature of more than 300 °C (above autoclave temperature). The designs were prepared in Autodesk Inventor and 3D printed by PCBWay 3D printing service. 1-4, Shows the assembly steps of the MEDIC in which a cooling fan with a 50 mm diameter was used to dissipate heat from the LEDs during the operation. 5-6, a Near-IR LED panel was installed, and then a 3D-printed thermocouple and PCR chip holder was tightened on the top. 7-8, Commercially available Dino-lite AM4117MT-G2FBW camera was used for the fluorescence image detection, mounted on the 3D printed camera holder. (**B**) Power supply TPP30-112A-J and 3D printed case. MEDIC uses ~8 watts of power, supplied by the 30 W power supply, approved for medical application.

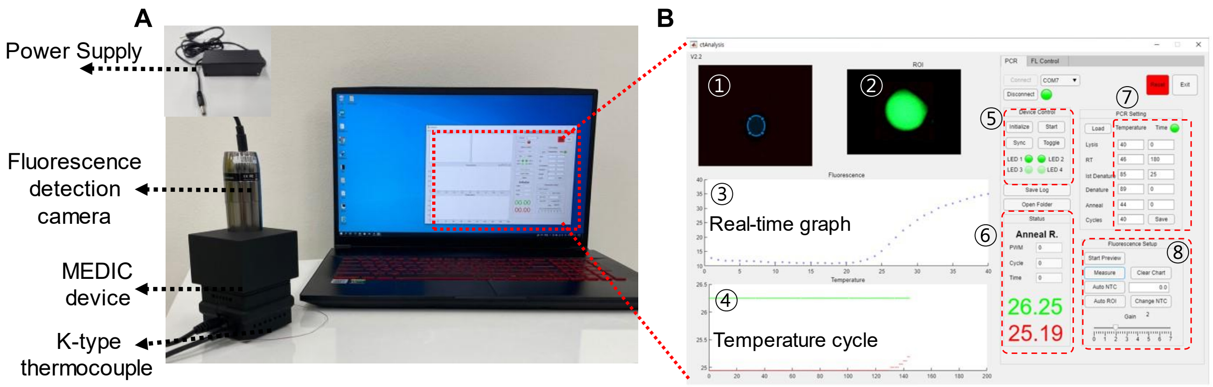


**Figure S10. Complete MEDIC setup and software.** (**A**) Fluorescence images were taken by Dino-lite AM4117MT-G2FBW camera on the MEDIC, interfaced to the computer via a micro USB cable, and the operation power was supplied by TPP 30-112A-J adapter housed in a custom-designed 3D case. (**B**) MATLAB was used to develop the software, including the image acquisition system to interface the camera. At the end of every annealing cycle, fluorescence images were taken. The image acquisition system shows the fluorescence preview aside from the fluorescent intensity curve of the PCR solution. The software also saved the recipe for different experiments, which can be reloaded quickly. The software also allows easy calibration in case of requiring recalibration. The software automatically determines the area of the PCR solution in the image to calculate the fluorescence intensity. Besides that, the software can save the temperature and fluorescence data logs. The fluorescence intensity signal was calculated from the images, captured automatically at the end of the annealing stage, and analyzed based on our image processing algorithm. The MEDIC device's RT-qPCR was configured using the software to amplify the SARS-CoV-2 N1 gene. The interface used a virtual serial port for transferring PCR configuration (recipes), data logs of temperature, and fluorescence intensities. The captured images were also taken and saved locally on the laptop: (1) Shows the real-time fluorescence preview whenever enabled by the user. (2) Shows the last captured fluorescence image during the thermal cycles. (3) The graph proceeds every thermal cycle, plotting the fluorescence intensity at the cycle. (4) The graph shows the solution temperature and room temperature from the secondary thermocouple, which can be used for calibration if required. (5) Control box to select the LEDs. (6) Current PCR information. (7) Temperature profile setting and assigning numbers for thermal cycles. (8) Camera and ROI configurations.

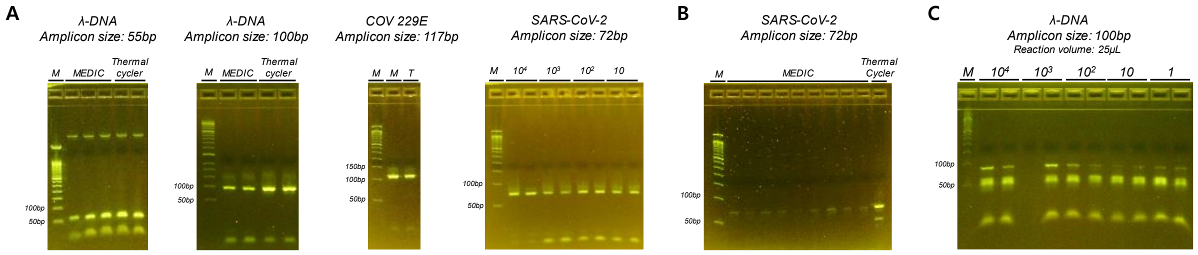


**Figure S11. Gel electrophoresis images of λ-DNA, COV 229E, and SARS-CoV-2 reference RNA compared with conventional RT-PCR and the MEDIC.** (**A**) Gel electrophoresis analysis with E-Gel$^{TM}$ power snap electrophoresis system (Invitrogen) showed results for testing λ-DNA, COV 229E and SARS-CoV-2 reference RNA using MEDIC, and the results are comparable to the commercial thermal cycler QuantStudio 3 real-time PCR instrument (Thermofisher). Lambda DNA (λ-DNA 250 µg/mL, 48/502 base pair) was used as template nucleic acid for the initial PCR test to corroborate the MEDIC. The DNA mix was prepared with KAPA2G fast DNA polymerase, which benefited us by reducing the total reaction time between 20% to 70% than those of conventional PCR assays performed with Taq DNA polymerase. (**B**) Consistency of the MEDIC was proved by testing 9 samples of SARS-CoV-2. **(C)** Gel electrophoresis image of the PCR result performed with MEDIC and 25 µL volume using a series of diluted samples ranging from $10^4$ copies per reaction to 1 copy per reaction.

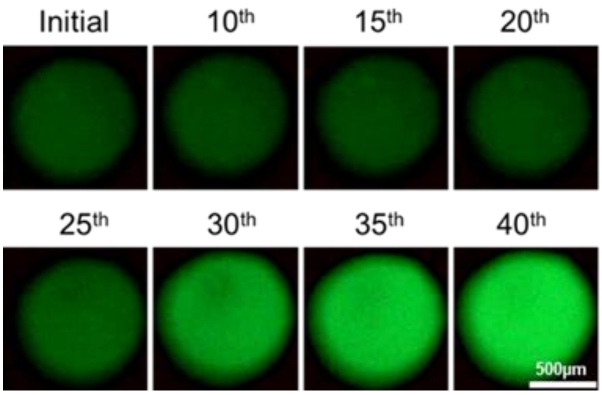


**Figure S12. Captured fluorescence images using real-time software.** Fluorescence images are taken every 5 cycles by the MEDIC-PCR.

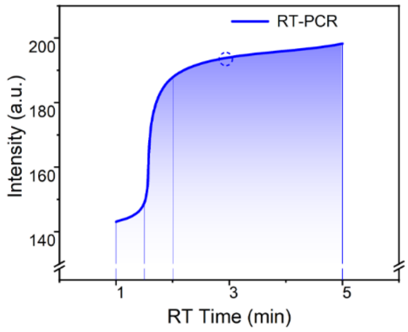


**Figure S13. Reverse transcription time optimization.** To optimize the reverse transcription time, we performed RT-PCR with different duration for reverse transcription and constant conditions for all the rest parameters. Then, we compared the final intensities for each duration. Since the curve started to saturate from 2.5 minutes, 3 minutes was selected as the optimized duration for the reverse transcription at 50 °C.

**A** 89 Positive samples

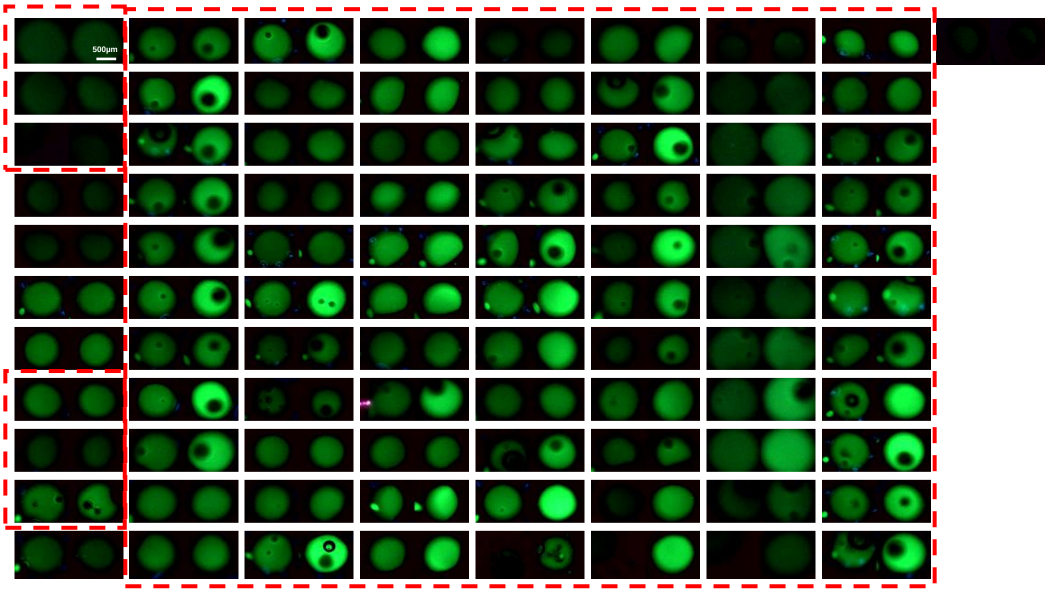


**B** 103 Negative samples

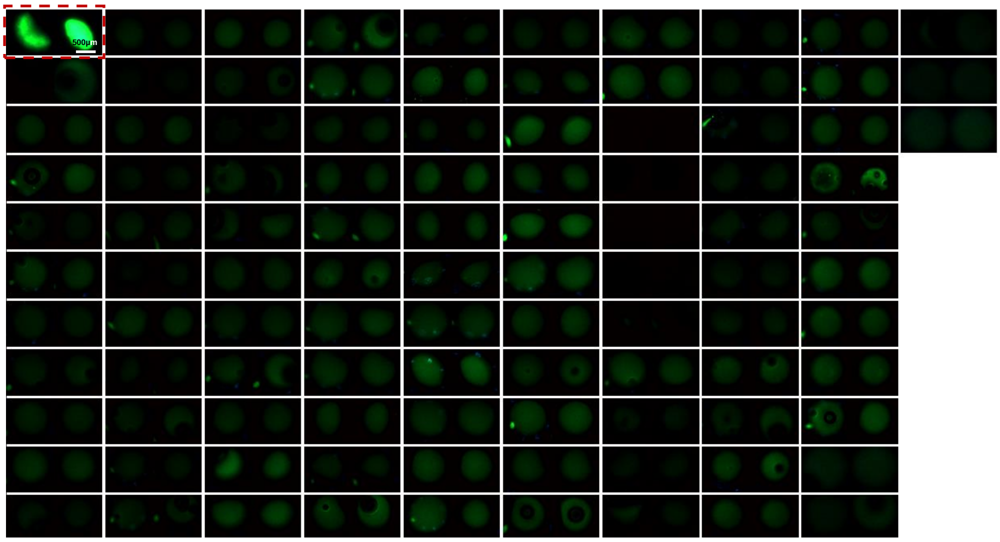

**Figure S14. Fluorescence images of clinical trials at the 1st cycle and 40th cycle.** (**A**) Fluorescence images at the first and last thermal cycle of 89 positive clinical samples, the red marked box represents 83 "true positive" results. (**B**) 103 negative clinical samples and one red-marked image showed the "false positive" results. The unusual fluctuation can be seen in the fluorescence curve due to the formation of bubbles in the fluorescence images. Such defects can be eliminated and completed in the future by the implementation of the advanced algorithm for fluorescence image processing.

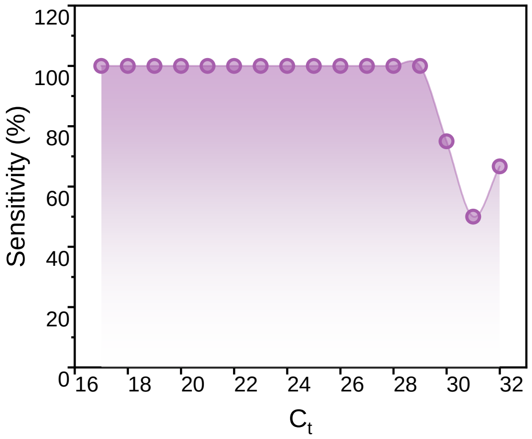


**Figure S15. Extended sensitivity of MEDIC.** The MEDIC can detect low viral concentration with standard PCR $C_t$ values as high as 32 from 1 µL of the PCR reagent. The sensitivity can be further improved by optimizing the lysis agent to run direct RT-qPCR.

**Note S1**

The simulation results verified the benefit of the inverted frustum to a cylindrical shape. The energy supplied to the device was calculated using the heat flux boundary condition, which estimated the flux from the power provided by the NIR (near-infrared) radiation. Natural convection aided in cooling down the chip after denaturing the cycle, and ambient boundary conditions were assumed for the surrounding air. The grid independence test was conducted to optimize the number of grid elements for both geometrical configurations, and it was observed that the cone-shaped structure responded better to the annealing and denaturing cycles, with higher heating and cooling rates.

The $k - \varepsilon$ model was used to simulate both the internal and two-phase flows, to predict the velocity and temperature distributions, as per Equation S2.

$$\frac{\partial(\rho k)}{\partial t} + \frac{\partial(\rho k u_i)}{\partial x_i} = \frac{\partial}{\partial x_j}\left[\frac{\mu_t}{\sigma_k}\frac{\partial k}{\partial x_j}\right] + 2\mu_t E_{ij}E_{ij} - \rho\varepsilon \qquad \text{(S2)}$$

$k$ = the turbulence kinetic energy
$\varepsilon$ = the rate of turbulent dissipation
$E_{ij}$= represents the component of the deformation rate
$\mu_t$ = represents the eddy viscosity
$u_i$ = represents the velocity component in the corresponding direction

Heat distribution is evaluated via the energy model, which predicts the heat fraction responsible for heating up/cooling down the device Equation S3.

$$\frac{\partial}{\partial t}(\rho E) + \nabla .\left(\vec{v}(\rho E + p)\right) = \nabla .\left(k_{eff}\nabla T - \sum_j h_j\vec{J}_j + \left(\overline{\overline{\tau_{eff}}}.\vec{v}\right)\right) + S_h \qquad \text{(S3)}$$

$k_{eff}$ = the effective thermal conductivity, i.e., k + $k_t$, where $k_t$ is the component due to turbulen ce
E = the total energy
$\vec{J_j}$ = the diffusion flux of component '$j$'
$\vec{v}$ = the kinetic energy component
$\overline{\overline{\tau_{eff}}}$ = represents the viscous dissipation
p = represents the work done by pressure
$S_h$ = represents the volumetric heat sources
